# FPGA-based digitizer for BGO-based time-of-flight PET

**DOI:** 10.1088/1361-6560/adc362

**Published:** 2025-03-28

**Authors:** Daehee Lee, Sun Il Kwon

**Affiliations:** Department of Biomedical Engineering, University of California, Davis, One Shields Avenue, Davis, CA 95616, United States of America

**Keywords:** bismuth germanate (BGO), coincidence timing resolution (CTR), time-to-digital converter (TDC), time-over-threshold (TOT), tapped delay line (TDL), Cerenkov (Cherenkov), time-of-flight positron emission tomography (TOF PET)

## Abstract

We present a novel field-programmable gate array (FPGA)-based bismuth germanate (BGO) time-of-flight (TOF) digitizer, implemented on an FPGA (XC7VX485T-2FFG1761C, Xilinx). This digitizer is designed to address the recently highlighted characteristics of BGO, which generates both scintillation and prompt Cerenkov photons when a 511 keV photon interacts with BGO. The developed digitizer independently processes these two types of photons for precise energy and timing measurements. The digitizer incorporates a noise-resistant binary counter that measures energy signals using the time-over-threshold (TOT) method. For timing measurements, we employ an embedded dual-side monitoring time-to-digital converter, which efficiently captures timing information while maintaining low resource usage. We validated the efficacy of our FPGA-based TOF digitizer through extensive experiments, including both electrical testing and coincidence measurements using BGO pixels. Our evaluations of TOT energy and timing performance utilized two 3 × 3 × 20 mm^3^ BGO pixels coupled to CHK-HD MT silicon photomultipliers. The digitizer achieved a coincidence timing resolution (CTR) of 407 ps full width at half maximum (FWHM) for events within the full width at tenth maximum of the photopeak in the measured TOT energy spectrum. Notably, when measured with an oscilloscope, the same detector pair exhibited a CTR of 403 ps FWHM, confirming that the performance of the developed digitizer is comparable to that of an oscilloscope. With its low resource usage, our design offers significant potential for scalability, making it particularly promising for multi-channel BGO-based PET systems.

## 1. Introduction

Bismuth germanate (BGO) initially served as a major scintillator in the formative stages of positron emission tomography (PET) detectors and is known for its excellent attenuation properties (Jones and Townsend 2017, Vandenberghe *et al* 2020, Yu *et al* 2022). Its widespread use underscored its importance within early PET systems. As the field advanced in the 2000s, the landscape of PET scintillators underwent a substantial transformation following the emergence of lutetium-based scintillators, including lutetium orthosilicate (LSO) and lutetium yttrium orthosilicate (LYSO) (Jones and Townsend 2017, Vandenberghe *et al* 2020). Given their superior light yield and shorter decay time, these LSO and LYSO scintillators gradually superseded BGO (Yu *et al* 2022).

As PET detector development has advanced, there has been growing interest in improving the signal-to-noise ratio of reconstructed PET images. This objective has been pursued through sophisticated methods to measure the arrival time difference between two back-to-back 511 keV photons within the sub-nanosecond range, enabled by improvements in signal measurement techniques. These developments led to the emergence of time-of-flight (TOF) PET, with lutetium-based scintillators becoming dominant in most commercial TOF PET systems due to their superior timing performance (Surti and Karp 2020). However, the higher cost of these materials compared to BGO has inevitably increased the overall expenses associated with PET system development (Yu *et al* 2022).

Recent studies on BGO, which is about three times cheaper than conventional TOF PET scintillators, reveal that approximately 17 prompt Cerenkov photons are generated when a 511 keV photon interacts with a BGO pixel, preceding scintillation photon emission (Brunner and Schaart 2017, Gundacker *et al* 2020). These Cerenkov photons are predominantly in the blue/UV range compared to scintillation photons (Kwon *et al* 2016, 2019, Trigila *et al* 2022). The significant enhancement in photon detection efficiency of silicon photomultipliers (SiPMs), particularly in the blue/UV range, has enabled the successful measurement of these few prompt Cerenkov photons in BGO (Kwon *et al* 2016, Kratochwil *et al* 2020). Notably, utilizing scintillation photons for energy measurement and prompt Cerenkov photons for timing measurement has substantially improved the coincidence timing resolution (CTR) to less than 500 ps (Cates and Levin 2019, Kwon *et al* 2019, Kratochwil *et al* 2021). These improved CTRs have opened up possibilities for developing TOF PET detectors using cost-effective BGO instead of the more expensive LYSO or LSO materials.

However, conventional integrated DAQ digitizer systems for TOF PET applications have been developed and optimized to acquire energy and timing information solely from the large number of scintillation photons produced by scintillators such as LYSO and LSO. Torres *et al* (2013), Son *et al* (2016), Ullah *et al* (2016), Lu *et al* (2019). These systems are designed to efficiently process large numbers of scintillation photons, making them less suitable for emerging BGO-based TOF PET systems, which require independent paths for energy measurement from scintillation photons and precise timing measurement from the significantly weaker Cerenkov photons (Piller *et al* 2024). To overcome these limitations, our field-programmable gate array (FPGA)-based approach introduces a novel architecture tailored for BGO-based TOF PET. Unlike conventional DAQ digitizers, our design incorporates a dual-side monitoring (DSM) TDC to significantly reduce timing measurement errors without requiring additional resource-intensive correction circuits (Lee *et al* 2024). Furthermore, we introduce a noise-resistant binary counter (NRBC) with double-check logic (DCL) to mitigate early termination artifacts in the TOT-based energy measurement, ensuring more accurate energy acquisition despite the relatively low number of detected photons in BGO. By independently utilizing Cerenkov photons for timing measurements and scintillation photons for energy measurement, our approach effectively enhances both energy and timing resolution, making BGO a more viable option for TOF PET applications.

In this research, we present a BGO TOF digitizer implemented on a FPGA, which offers rapid design capabilities and reprogrammable circuit flexibility (Sohal *et al* 2014, Machoado *et al* 2019). This study examines each critical component for energy and timing measurement, and introduces the comprehensive system developed for BGO-based TOF PET modules. We also describe an experimental setup used for extensive electrical testing and coincidence measurement. Finally, we present test results obtained using 3 × 3 × 20 mm^3^ BGO pixels coupled to CHK-HD MT SiPMs (FBK, Italy). CHK-HD MT SiPM was chosen for its high detection efficiency in the Cerenkov wavelength range and its ability to operate at higher bias voltages, improving timing resolution. Gundacker *et al* (2023), Merzi *et al* (2023).

## 2. Material and methods

### 2.1. Energy and timing signals from BGO pixels

**Figure 1.**
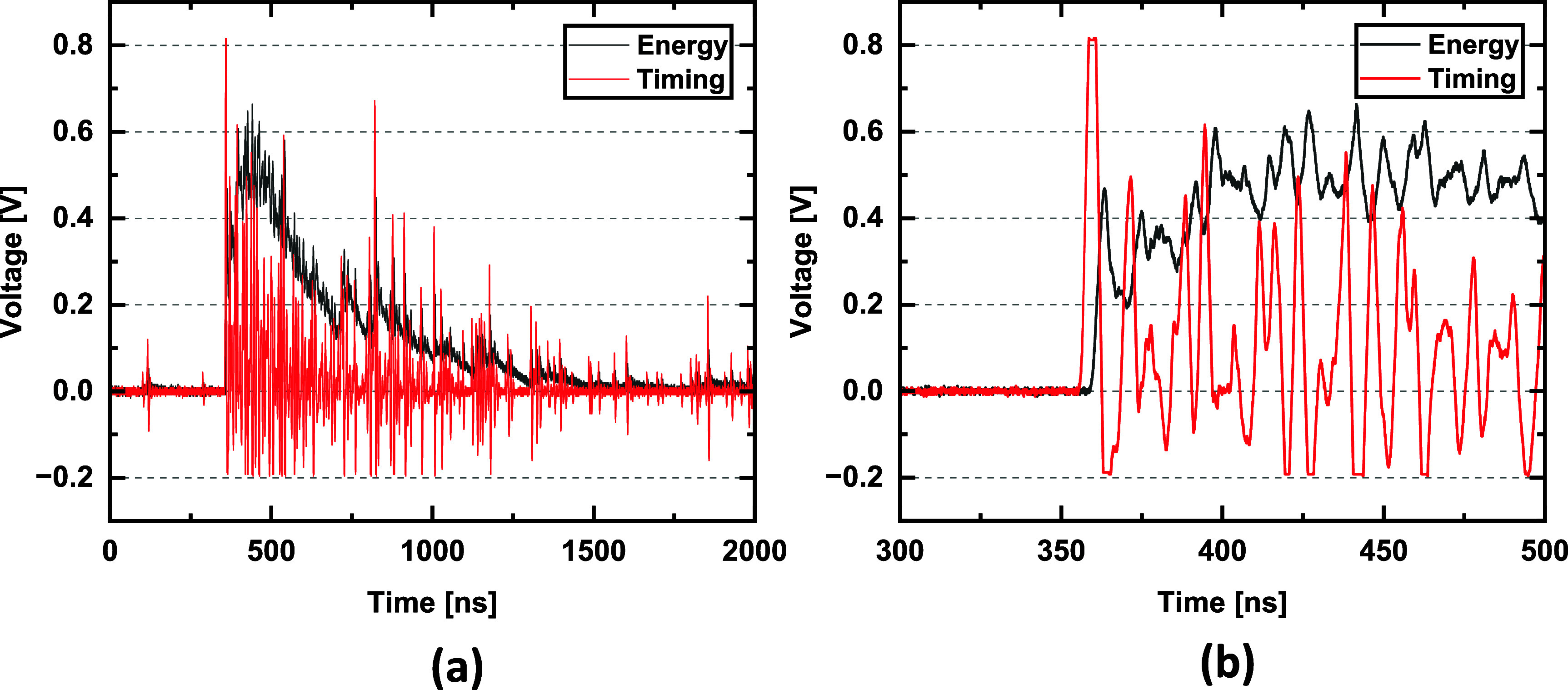
(a) Energy and timing signals of 3 × 3 × 20 mm^3^ BGO pixels coupled to CHK-HD MT SiPMs. (b) Enlarged views of the rising parts of the energy and timing signals, respectively.

The BGO pixels were coupled to CHK-HD MT SiPMs and pole-zero cancellation circuits, which generated the energy and timing signals (Gola *et al* 2011, Gundacker *et al* 2023). To verify the signal properties, we digitized signals using an oscilloscope (MSO64B, Tektronix) with a bandwidth of 4 GHz and a sampling rate of 25 GS s^−1^. Both CHK-HD MT SiPMs, with a breakdown voltage of 32.5 V, were subjected to a bias of 49 V throughout the experiments, except during a bias optimization test. Figure 1(a) presents typical energy and timing signals acquired using two 3 × 3 × 20 mm^3^ BGO pixels (Epic Crystal, China) and a ^22^Na point source for 2 *μ*s. They are presented here preemptively as they directly illustrate the key signal characteristics that support the proposed method. Notably, figure 1(b) clearly shows spike-shaped signals during energy measurements, produced by the fast signal component of a single fired cell in a SiPM (Gundacker *et al* 2023, Merzi *et al* 2023). Depending on the measurement method, particularly in the time-over-threshold (TOT) method, these spike signals may cause early termination of the energy measurement process, making accurate energy measurement difficult. This issue is discussed in more detail in the following section. The energy signal is slower than the timing signal is due to the offset time delay caused by the difference in amplifiers for generating the energy signal and the timing signal.

### 2.2. Energy measurement unit

The proposed FPGA-based digitizer employed the TOT technique as an energy measurement unit due to its efficient use of the FPGA’s inherent digital components: a binary counter (BC) and a comparator (Won *et al* 2020, 2021). The TOT method, common in particle physics and imaging fields, allows for the transformation of energies into digital values in a resource-effective way (Jakubek 2011, Chang *et al* 2017, Urban *et al* 2023).

**Figure 2.**
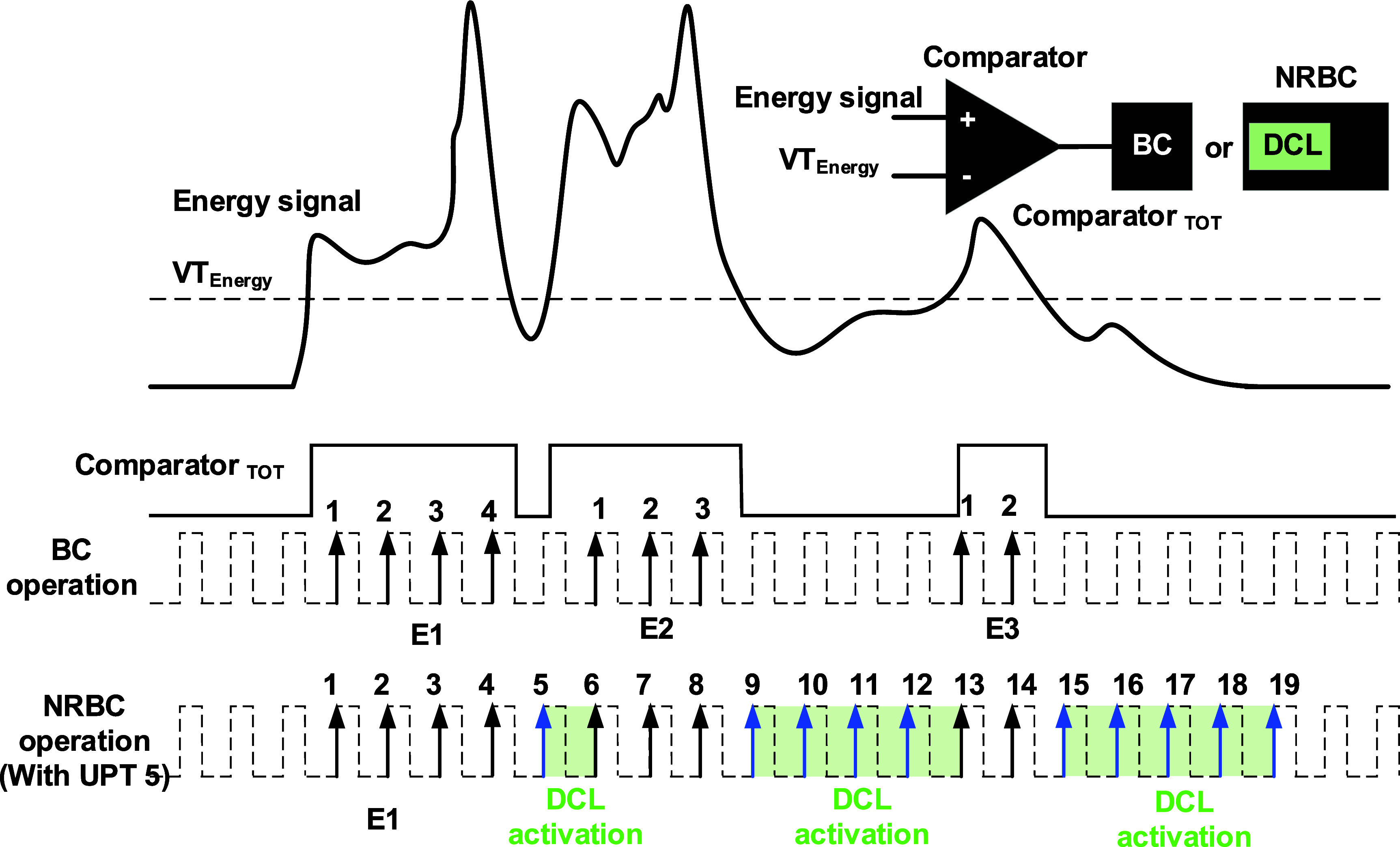
TOT energy measurement operation using binary counter (BC) and noise-resistant BC (NRBC).

The TOT method involves establishing a predetermined threshold (*VT*_Energy_) for one of the comparator’s inputs, as shown in figure 2. The subsequent BC quantifies the high state’s duration of comparator output (Compartor_TOT_), which surpasses this threshold. The BC TOT value is incremented in synchronization with a system clock while Comparator _TOT_ is in a high state, thereby signifying the energy of the input signal. For instance, with a system clock of 550 MHz (1.8 ns clock period), an input energy signal with a duration of 4 ns is translated into a BC TOT value of #2, representing an energy signal range from 3.6 ns (1.8 ns × #2) to 5.4 ns (1.8 ns × #3). Although a faster clock frequency enables more precise TOT energy measurements, power consumption also increases significantly. Therefore, the system clock frequency should be optimized for each dedicated system (Drozd *et al* 2021). In addition, the recorded TOT values may exhibit distortions in the high energy region depending on the threshold level, indicating the necessity for optimal threshold voltage selection for the TOT spectrum (Jakubek 2009, 2011).

Managing local spikes in energy signals with the conventional TOT method, as depicted in figure 1(b), presents a significant challenge for the BC. Local spikes on the rising phase of the energy signal could cause multiple transitions at the comparator’s output, potentially prematurely terminating the BC operation and recording multiple lower TOT values instead of the correct higher TOT energy value, as explained in figure 2. To address this issue, we proposed a NRBC incorporating a DCL. The NRBC design, illustrated in figure 2, uses the DCL to prevent premature terminations caused by local spikes. When the comparator’s output transitions to low, the DCL monitors for any high-state regeneration within the user-defined period time (UPT). If the signal reappears before the UPT completes, the BC TOT operation continues, ensuring accurate TOT measurements.

Without NRBC, the system would prematurely record lower TOT values, leading to inaccurate coincidence event acquisition. Ultimately, this reduces data acquisition efficiency and degrades the overall energy spectrum. The NRBC sets the UPT value as the minimum TOT threshold once energy measurement begins, preventing erroneous terminations.

### 2.3. Timing measurement unit

**Figure 3.**
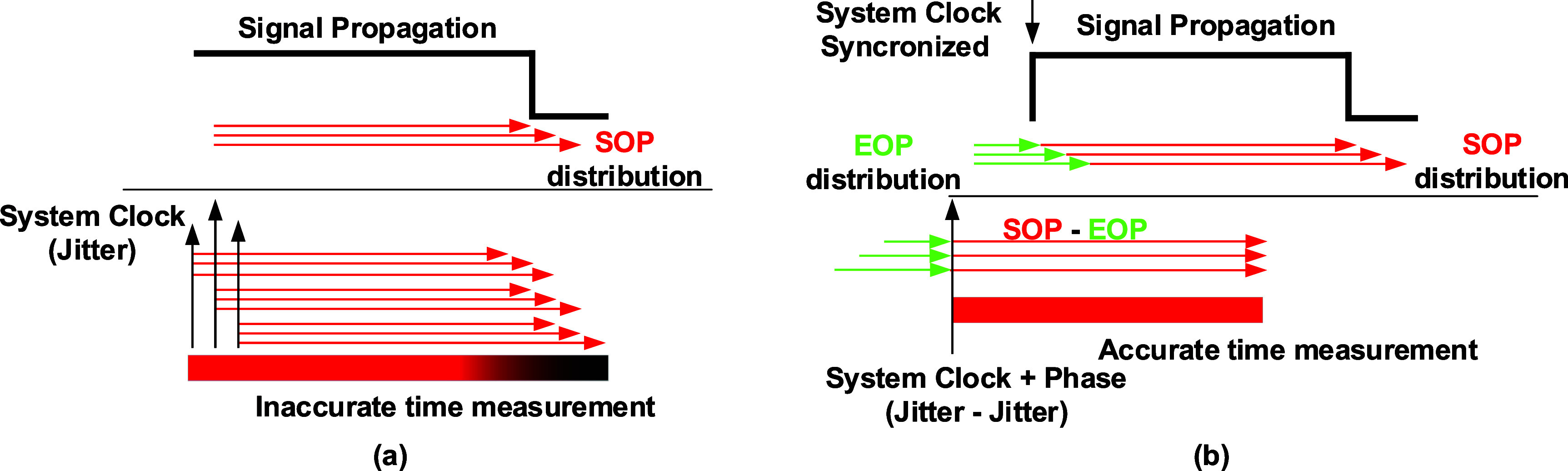
Comparison of time measurement methods: (a) conventional method focusing solely on start of propagation (SOP), and (b) proposed DSM TDC method incorporating both SOP and end of propagation (EOP).

Accurate timing measurement is essential for coincidence detection in TOF PET systems, and time-to-digital converters (TDCs) play a crucial role in precisely determining the arrival-time differences between coincident events. Conventional FPGA-based TDCs primarily utilize delay lines or multiple delay lines (CARRY4 chains) for picosecond-scale time measurements, typically relying solely on start of propagation (SOP) detection (Wang *et al* 2018, Qin *et al* 2020, Szplet and Czuba 2021, Kim *et al* 2023). As illustrated in figure 3(a), this approach measures the signal’s arrival time based only on SOP, where the CARRY4 structure, consisting of four internal delay elements with an average propagation delay of approximately 10 ps per element, is commonly used in high-precision TDC designs as demonstrated in previous studies using Virtex-6 and Kintex-7 FPGAs (Won and Lee 2016).

However, SOP-only TDCs are highly sensitive to power–voltage–temperature (PVT) variations, as shown in figure 3(a). Since the signal propagation speed depends on these factors, additional calibration and correction circuits are required, increasing FPGA resource consumption and circuit area. Furthermore, clock jitter introduces additional measurement uncertainty, demanding further resource-intensive corrections (Won and Lee 2016, Machado *et al* 2019). These limitations pose a significant challenge in large-scale TOF PET systems, where numerous detector channels must be implemented within a limited FPGA fabric.

To overcome these issues, we employed a DSM TDC (Lee and Kwon 2024). As illustrated in figure 3(b), the DSM TDC monitors both the SOP and the end of propagation (EOP) signals. This dual monitoring approach ensures that SOP and EOP share comparable propagation characteristics, significantly reducing errors caused by clock jitter and PVT variations. Unlike conventional TDCs, which require additional resource-intensive correction circuits, the DSM TDC improves timing accuracy while minimizing FPGA resource usage (Lee and Kwon 2024).

### 2.4. Two controllers for BGO TOF digitizer operation

**Figure 4.**
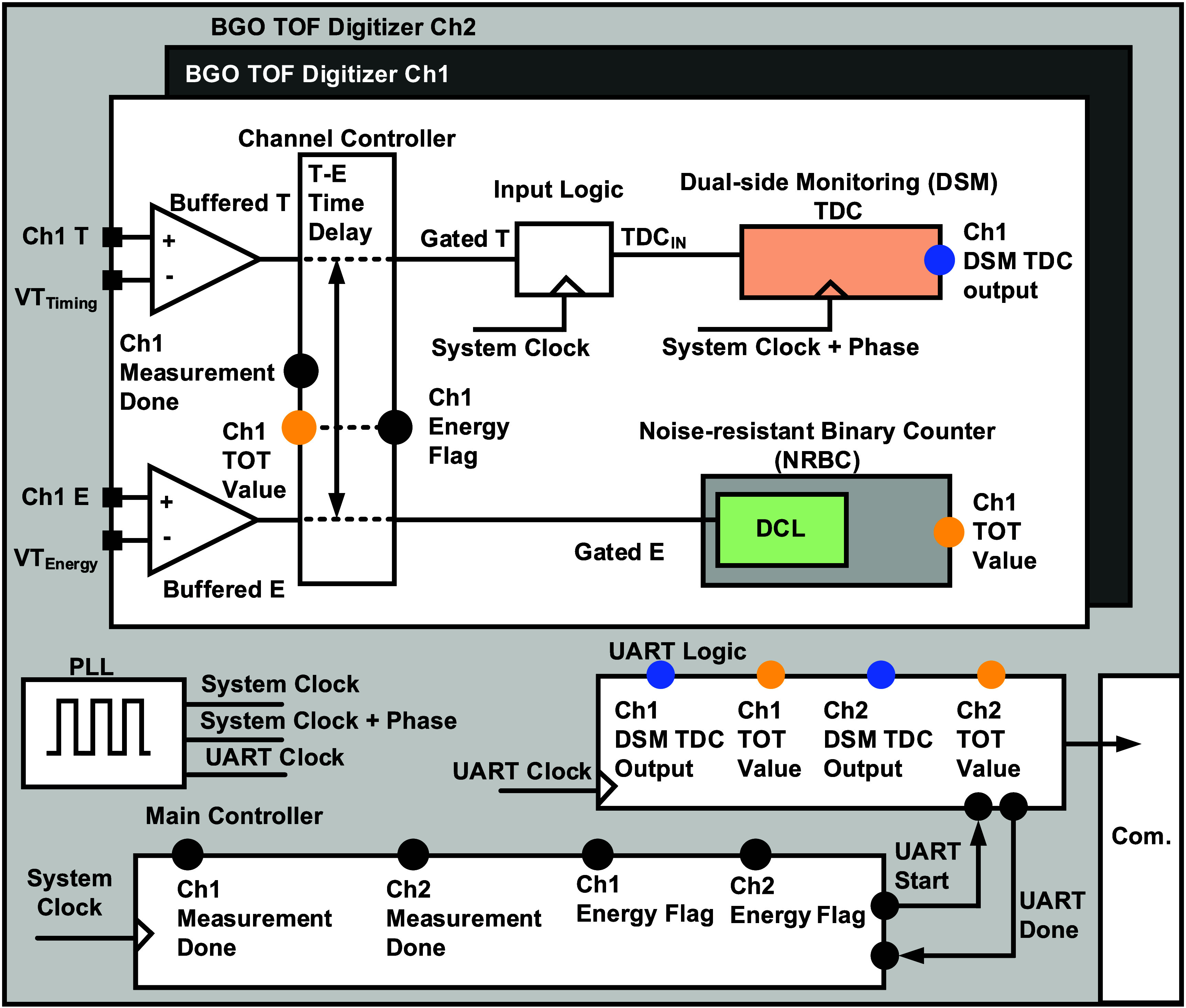
Block diagram of the proposed BGO TOF digitizer with peripheral units.

The BGO TOF digitizer consists of a channel controller and a main controller, both of which work together to process energy and timing signals while ensuring accurate coincidence event detection. The overall system architecture is illustrated in figure 4, which shows the functional blocks and their interconnections within the digitizer.

The channel controller in the BGO TOF digitizer initially inspects *Buffered T* (timing) and *E* (energy) signals, which are the outputs of a low voltage differential signaling (LVDS) buffer for the timing and energy inputs, respectively. The *Gated T* and *E* signals are activated when the *Buffered T* and *E* signals are stable, as controlled by the channel controller. These gated signals are subsequently used for timing and energy measurements in the DSM TDC and NRBC, respectively.

Following the gating process for the timing and energy signals, the channel controller verifies the time difference (*T–E Time Delay*) between the *Gated T* and *E* signals for each channel input. This enables the rejection of timing signals inaccurately triggered by noise. If the timing signal is detected earlier than the noise rejection window from the energy signal input, the channel controller resets its channel to repeat the signal check stage for *Buffered T* and *E*. An optimal noise rejection window is determined based on experimental results.

The channel controller further examines the measured TOT value to verify if it exceeds a predefined cut-off TOT value. This examination is essential to reject events triggered by transient spike signals from the SiPM detector. The processed results, labeled as *Energy Flag*, are subsequently forwarded to a main controller. The main controller then determines if each collected coincidence event from two channels is valid.

**Figure 5.**
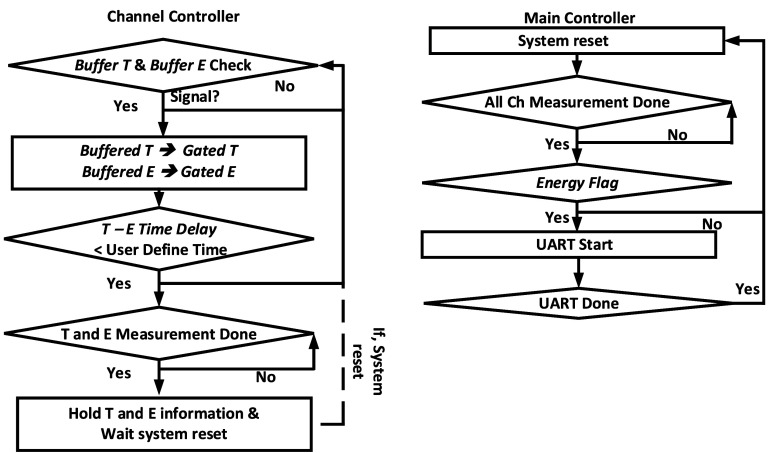
Operating flowchart for both the channel controller and the main controller.

The main controller, located outside the BGO TOF digitizer modules, is responsible for managing multiple BGO TOF digitizer channels and determining whether both energy signals surpass the predetermined minimum TOT value. This information is communicated through the *Energy Flag* from each channel controller. If *Energy Flag*s from both channels register as high, all recorded values (including TDC outputs, coarse counters, and TOT values) from each channel are transmitted to a computer via the universal asynchronous receiver-transmitter (UART) logic. Figure 5 illustrates the operational procedures of both the main and channel controllers.

The system clock for the BGO TOF digitizer operates at 550 MHz, with one clock cycle (1.8 ns) covering 44 CARRY4s in a delay line (43 CARRY4s for SOP and an CARRY4 for EOP) within the same clock region (X0Y0). The frequency of 550 MHz was chosen as the maximum feasible frequency to form a delay chain within a single clock region, ensuring precise and efficient time measurement (Won and Lee 2016). To optimize power consumption, the UART logic operates at a significantly lower frequency of 55 MHz (UART clock). To apply consistent effective threshold voltages for both energy and timing signals, we utilized LVDS input protocols and corrected for offset differences in these signals due to circuit variations.

## 3. Experimental setup

**Figure 6.**
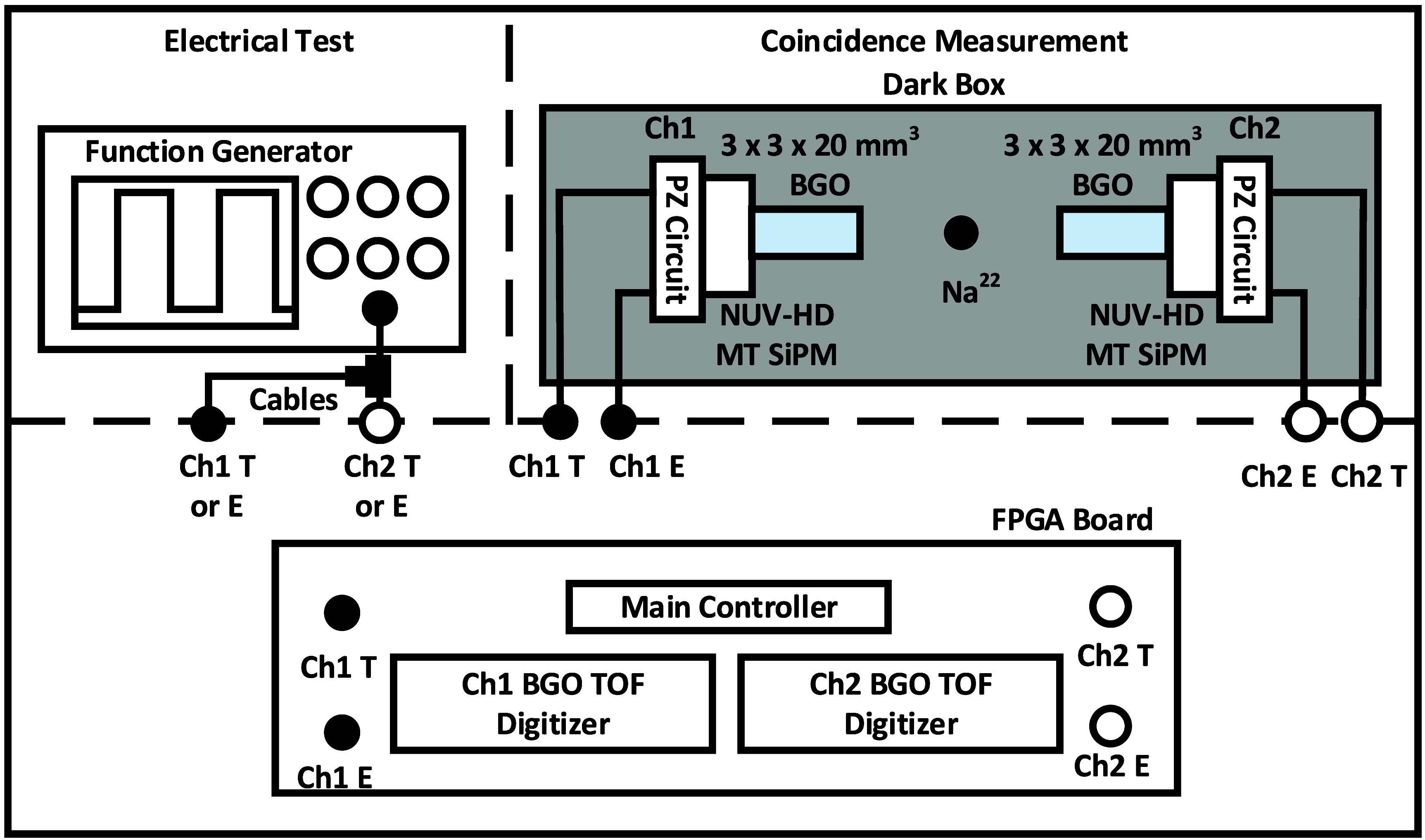
Experimental setup for electrical tests and coincidence measurement with 3 × 3 × 20 mm^3^ BGO pixels.

NRBC and DSM TDC functionalities in the BGO TOF digitizer were evaluated using an electrical setup that utilized a single output from a function generator (SDG6052X, Siglent Technologies), as shown in figure 6. The evaluation process consisted of two main phases: NRBC and DSM TDC functionality assessments with the electrical test setup.

For the NRBC functionality evaluation, the function generator’s output, square pulses, was split via a *T*-connector, with both signals fed into the Ch1 E and Ch2 E ports on the FPGA board. The pulse width varied from the generator’s minimum value (4 ns) up to the measurable counter value, #1000 for NRBC in this study. The NRBC featured its in-built DCL and an offset minimum counter value determined by the UPT. The NRBC’s maximum count value was set to #1,000, enabling a measurable count range from # UPT to #1000.

In assessing the TDC functionality, time delays between Ch1 T and Ch2 T were introduced by copying the same signal using five different cable combinations: Cable A-B, A-C, A-A′, B-A′, and C-A′. A and A′ are cables of identical length, with A′ designated to differentiate between the two. These intervals were strategically chosen to validate the TDC’s performance both within and beyond a single system clock period (1.8 ns) and were cross-verified using the oscilloscope. Time differences within the system clock period of 1.8 ns were generated with three different cable combinations: cables A-B, A-A′, and B-A′. Time differences that exceeded a single system clock cycle were produced using cables A-C or C-A′. The intervals, marked as differences between Ch1 T and Ch2 T, were set to 2,050 ps (Cable A-C), 260 ps (Cable A-B), 0 ps (Cable A-A′), −260 ps (Cable B-A′), and −2050 ps (Cable C-A′).

Throughout these tests, the square pulse output of the function generator was maintained at a consistent amplitude of 1 Vpp. This resulted in an effective 500 mV applied to all negative LVDS inputs (*VT*_Energy_ or *VT*_Timing_), as demonstrated in figure 6. During these experiments, the NRBC or DSM TDC modules were deactivated to assess the independent functionality of each module.

The CTR measurement involved two identical BGO pixels placed inside a dark box, each coupled with a CHK-HD MT SiPM. Both timing (Ch1 T and Ch2 T) and energy signals (Ch1 E and Ch2 E) were connected to each BGO TOF digitizer. To compensate for the offset voltage differences across energy and timing signals, individual threshold voltages were applied to the negative inputs of the LVDS buffers. For optimal CTR, the *VT*_Timing_ was set slightly above the noise level (Kwon *et al* 2016, 2019). A *VT*_Timing_ of 4 mV was applied across all timing channels and remained fixed for all subsequent experiments.

Regarding *VT*_Energy_, an optimum UPT for NRBC operation was explored by applying BGO energy signals to the BGO TOF digitizer. The jitter at *Gated E* caused by local spikes in the rising part of the energy signals was investigated, and the optimum UPT was selected for stable TOT energy acquisition. Effective *VT*_Energy_ values of 50 mV, 100 mV, 150 mV, 200 mV, and 250 mV were investigated to optimize the TOT energy performance for enhanced timing performance.

## 4. Results and discussion

### 4.1. Evaluation of the NRBC functionality

**Figure 7.**
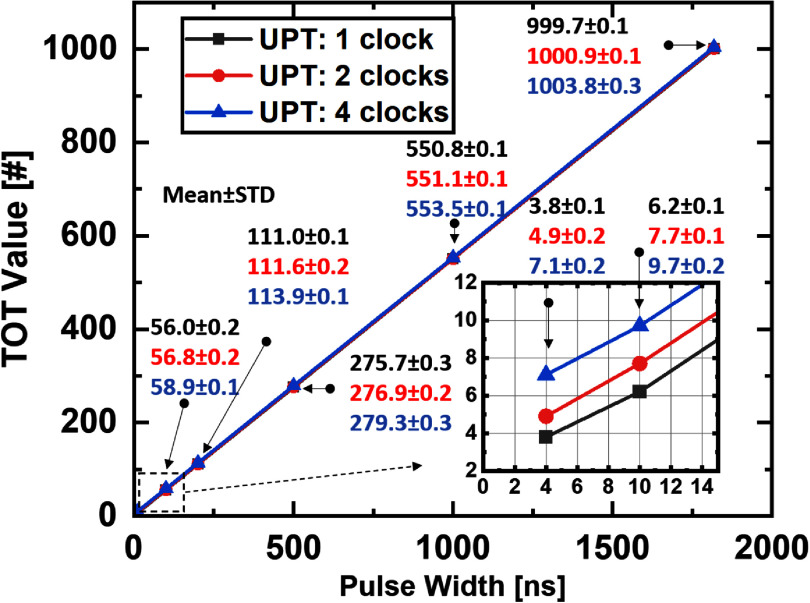
Average TOT values measured by the NRBC using three different user-defined period times (UPTs): 1, 2, and 4 clocks. The enlarged view highlights detailed variations in measurements.

Throughout the electrical experiments, the input pulse width was measured by the NRBC, utilizing the function generator output. Square Pulse widths of 4, 10, 100, 200, 500, and 1800 ns were tested, while the square pulse period was maintained at a constant 3 *µ*s. Start points for measuring TOT values at the BGO TOF digitizer were randomized due to the asynchronization between the 550 MHz system clock for the BGO TOF digitizer and the input frequency from the function generator. As a result, variations were observed in the measured TOT values. For each pulse width setting, 1000 samples were collected, and the TOT values were then averaged, as depicted in figure 7. For instance, the measured average TOT values were #3.8 ± 0.1, #4.9 ± 0.2, and #7.1 ± 0.2 for 4 ns pulse width inputs with 1, 2, and 4 clock UPT values, respectively.

Figure 7 shows the average TOT values for six distinct input pulse widths with three different UPT conditions, with standard deviations not exceeding #0.3. As shown in an enlarged graph in figure 7, different UPT values resulted in offset counter values for each UPT setup. However, these offsets did not affect the linearity of the NRBC, as all calculated R-squared values exceeded 0.9999. The robust performance of the NRBC was confirmed across all UPT setups, demonstrating its superior linearity throughout the entire measurable TOT range. Furthermore, CTR measurements conducted with the SiPM biased at 49 V and *VT*_Energy_ set to 250 mV yielded values of 452 ps, 431 ps, and 407 ps for 1-, 2-, and 4-clock UPT values, respectively. These results indicate that lower UPT values lead to CTR degradation, as the NRBC operation gradually converges toward that of a conventional BC, resulting in premature termination of energy measurement.

### 4.2. Evaluation of the DSM TDC functionality

**Figure 8.**
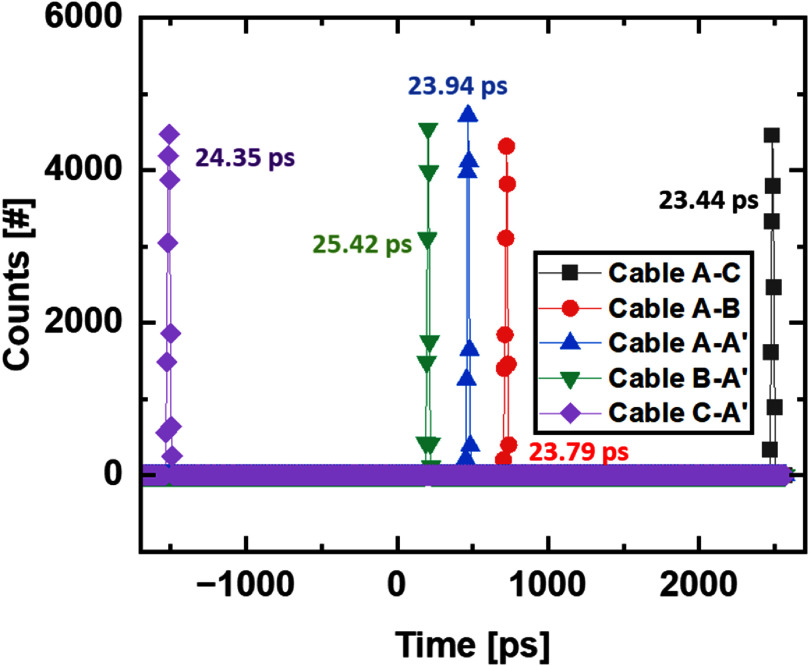
Measured time intervals using the DSM TDC. FWHM values for each measurement are indicated.

Histogram data of each measured time difference were fitted with a Gaussian curve to calculate the full width at half maximum (FWHM) value. A time bin of 5 ps was used for the histogram plot, providing a good fit across all measured points. Figure 8 displays the FWHM of the peak value alongside corresponding time measurements. For the 0 ns (Cable A–A′) input, the offset time difference between Ch1 T and Ch2 T was approximately 480 ps, due to internal routing differences from an input pad to an input logic component, which is the first component for the DSM TDC. Although this offset could be corrected by subtracting the offset time difference from the result, the original data were analyzed in the current stage to present the unmodified information. Notably, the DSM TDC accurately identified all five time-intervals, exhibiting an average FWHM of 24.19 ps. Meanwhile, the oscilloscope achieved an average FWHM of 30.12 ps for the same input data.

To evaluate the linearity of the DSM TDC, the centers of each Gaussian fit were used in the linearity calculation. The calculated linearities, as indicated by the R-squared values, were 0.9997 for the DSM TDC and 0.9889 for the oscilloscope. Thus, the DSM TDC demonstrated not only high levels of accuracy but also notable linearity. The comparatively lower performance of the oscilloscope might have resulted from its limited sampling frequency of 25 GS s^−1^. These findings emphasize the effectiveness of the DSM TDC for precise time interval measurements.

### 4.3. TOT energy spectra with BGO pixels

An optimal UPT value is of critical importance for the NRBC, as it is directly linked to the termination of each TOT measurement at the BC by the channel controller. This termination occurs when jitters are introduced at *Gated E* due to the crossing action between local spikes and *VT*_Energy_ at the LVDS buffer. Hence, eliminating any jitter on *Gated E* is imperative for obtaining the correct TOT energy of each photon interaction event. For practical BGO applications, an optimal UPT for the NRBC was determined based on the pulse widths of the jitter signals.

**Figure 9.**
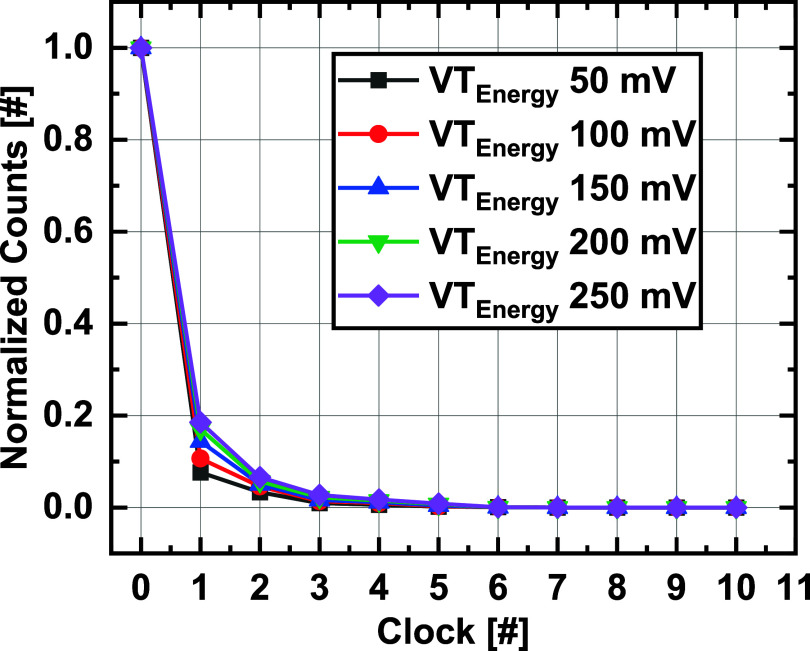
Distribution of measured pulse width jitters at *Gated E*, caused by local spikes.

The coincidence experimental setup was employed to monitor *Gated E* signals from an actual BGO pixel. The *Gated E* signal was routed to an external oscilloscope from the FPGA. An investigation was carried out into the pulse widths of the jitter at *Gated E* with five different *VT*_Energy_ values. Three thousand samples were collected for each *VT*_Energy_. The measured pulse widths were histogrammed and normalized in figure 9, as a function of the system clock period (1.8 ns). Values at one and two clocks decreased as the *VT*_Energy_ threshold increased due to multiple pulses at *Gated E* being triggered by local spikes. Notably, none of the jitter pulse widths exceeded 5 clocks for all *VT*_Energy_ values. Based on these results, a decision was made to select 4 clocks as an optimal UPT value. This selection established a minimum countable TOT value of five in the NRBC for all subsequent experiments discussed in this study.

**Figure 10.**
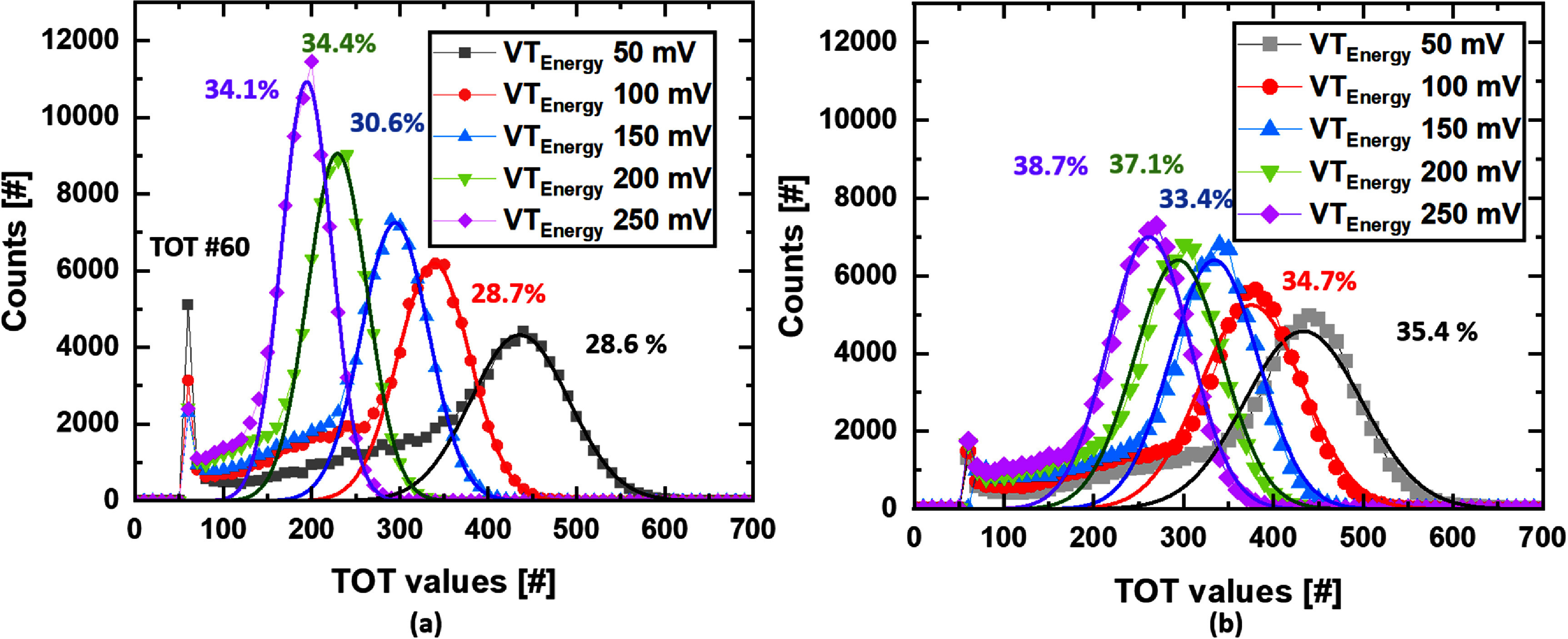
Total energy spectra measured at bias voltages of 49 V (a) and 51 V (b). In (a), spectra are shown for five different *VT*_Energy_ thresholds, with Gaussian fits applied to each. The calculated energy resolutions are indicated next to the corresponding fitting results.

By applying the optimal 4-clock for UPT, events surpassing a TOT value of #60 were processed by both Ch1 and Ch2 channel controllers. The cut-off TOT value of #60 was chosen to ensure efficient DAQ while still capturing the 511 keV peak without compromising the analysis. Thus, the #60 threshold was set in this study to mitigate these effects. Apart from the electrical test, the NRBC was operated with the DSM TDC for a coincidence experiment, demonstrating the full operation of the BGO TOF digitizer. For the TOT energy spectra, a total of 90 900 events for each *VT*_Energy_ threshold were collected by the NRBC. The acquired TOT energy spectra were plotted using a bin size of 10 TOT values for two different bias voltages of 49 and 51 V in figure 10. Owing to the applied cut-off TOT value (#60) at the channel controller, zero counts were observed below #60 TOT values in the TOT spectra. Higher values around the #60 TOT value were attributed to system noise.

Each 511 keV peak in the TOT energy spectra was fitted with a Gaussian function. No TOT energy distortion corrections were applied to the energy spectra presented in figure 10, resulting in the absence of a clearly defined Compton region (Sharma *et al* 2020). It has been previously reported that the Compton scattering region is not well distinguished in TOT-based energy measurements (Grant and Levin 2014, Nadig *et al* 2023). In a previous study on LYSO, the Compton region became less distinguishable at higher bias voltages when TOT energy measurements were derived from the timing signal. However, in our experiment using BGO, where the energy signal was used for TOT measurements, the Compton region was not clearly observed even at lower voltage levels. Energy resolutions were calculated using the FWHM values of the Gaussian fits for each case.

For the bias voltage of 49 V in figure 10(a), as *VT*_Energy_ increased from 50 mV to 250 mV in 50 mV step, a degradation trend was observed in energy resolution, displaying 28.6%, 28.7%, 30.6%, 34.4%, and 34.1%, respectively. Further increase in *VT*_Energy_ was not indicated as the 511 keV photo peak neared the cut-off TOT (#60) value in our setup. A Compton edge was not distinctly visible in the TOT energy spectrum across all energy spectra. For optimum timing calculations, the data within the full width at tenth maximum (FWTM) value of each Gaussian fit for each spectrum were utilized.

Similarly, for the different bias voltage of 51 V in figure 10(b), the energy resolutions were calculated as 38.7%, 37.1%, 33.4%, 34.7%, and 35.4% for *VT*_Energy_ values of 50 mV, 100 mV, 150 mV, 200 mV, and 250 mV, respectively. The degradation trend in energy resolution with increasing *VT*_Energy_ remained consistent at 51 V but showed slightly higher absolute values than at 49 V. This decline is likely due to increased SiPM dark noise at higher bias voltages, which adds noise to the energy spectrum and reduces measurement precision (Kang *et al* 2015). The absence of a clearly defined Compton edge was also observed in the 51 V spectra, consistent with previous findings (Nadig *et al* 2023). The impact of *VT*_Energy_ on CTR will be discussed in the next chapter.

### 4.4. Timing performance with BGO pixels

**Figure 11.**
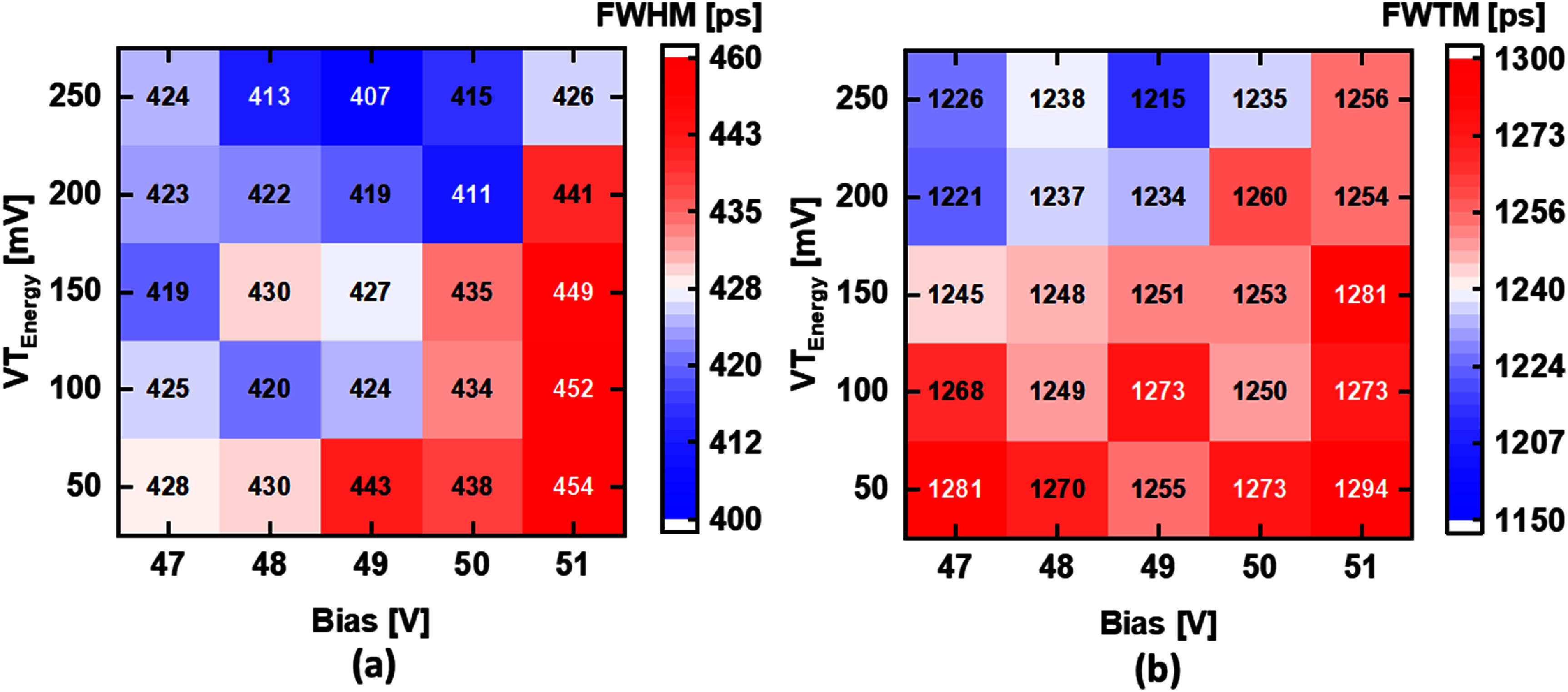
FWHMs (a) and FWTMs (b) in CTRs for the bias and *VT*_Energy_ sweeps measured using the developed BGO TOF digitizer.

In pursuit of the optimal CTR condition, *VT*_Energy_ thresholds for TOT energy spectra and bias voltages for CHK-HD MT SiPMs were systematically investigated, with *VT*_Timing_ maintained at 4 mV. Figure 11 illustrates the CTRs obtained with various *VT*_Energy_ thresholds and bias voltages. *VT*_Energy_ thresholds varied from 50 mV to 250 mV in 50 mV increments, while the bias voltage was adjusted from 47 V to 51 V in 1 V steps. Intriguingly, no clear correlation between energy resolution and timing performance was observed. For instance, a better energy resolution (28.6%) with a *VT*_Energy_ of 50 mV resulted in a 440 ps FWHM in CTR, while a worse energy resolution (34.1%) with a *VT*_Energy_ of 250 mV yielded better timing performance with a 407 ps FWHM in CTR, when a 49 V bias was applied to both CHK-HD MT SiPMs. This discrepancy is likely related to TOT energy distortion, though no corrections were applied in this study. The observed improvement in CTR may result from the higher *VT*_Energy_ threshold filtering out weaker signals and enhancing timing precision. A more precise evaluation would be appropriate after applying TOT energy distortion corrections using the dual TOT method, as discussed in section 4.5.

In the experimental results, the optimal CTR was achieved with a 49 V bias voltage and a *VT*_Energy_ threshold of 250 mV. Both FWHM and FWTM values were optimal among the 25 different setups used for CTR calculations.

**Figure 12.**
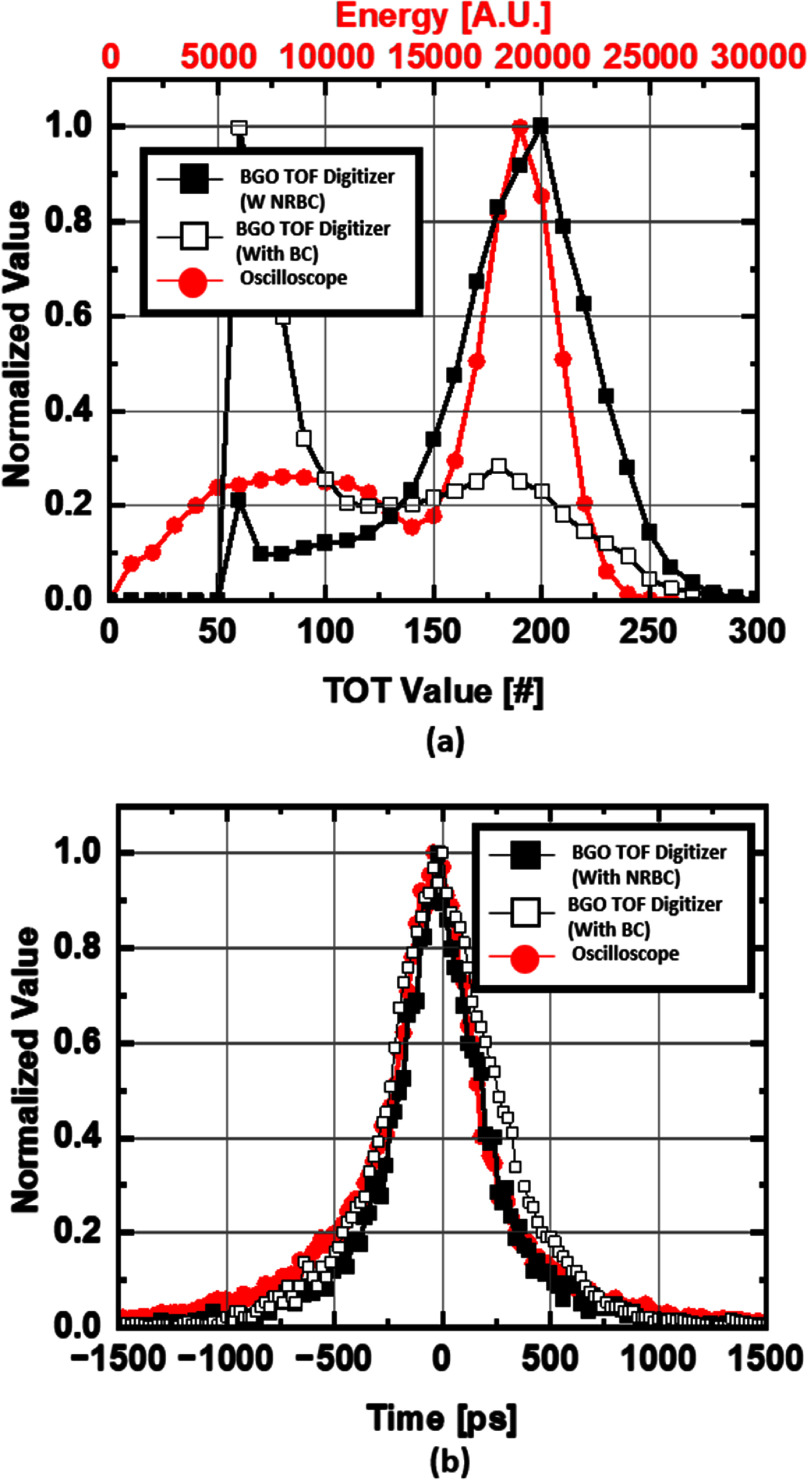
Energy spectra (a) and timing spectra (b) for the oscilloscope and the BGO TOF digitizer (with BC and NRBC) measured at a SiPM bias of 49 V and *VT*_Energy_ of 250 mV. The same experimental setup was used to minimize variations due to changes in the experimental configuration.

Figure 12 provides a comparison of the energy spectra and coincidence timing spectra for the BGO TOF digitizer with BC and NRBC operation, as well as the oscilloscope, using the experimental setup that yielded the best CTRs for the BGO TOF digitizer. A total of 90 900 events were collected and analyzed in each case.

The energy spectra for the systems are presented in figure 12(a). The integration-based energy spectrum was obtained by summing each energy signal over its time interval as measured by the oscilloscope. Since the integration-based energy spectrum for the oscilloscope data is in a different unit than the TOT value for the BGO TOF digitizer, two different *x*-axes were used: the bottom *x*-axis for TOT values and the top *x*-axis for integration values. A Compton edge was clearly observed in the integration-based energy spectrum. The energy spectra obtained from the BGO TOF digitizer using both BC and NRBC methods are also presented in figure 12(a). The BC-based measurement exhibits an abnormally high event count around the TOT value of #60, indicating premature termination of the TOT measurement, as discussed in figure 2. Consequently, the 511 keV photopeak does not correspond to the highest event count, leading to an energy resolution of 79.1%. In contrast, the energy spectrum measured using the NRBC method achieved an energy resolution of 34.1%, as calculated by Gaussian fitting of the 511 keV photopeak. For reference, the oscilloscope-based integration method yielded a superior energy resolution of 21.4%. This discrepancy in energy resolution arises from fundamental differences between the TOT and integration-based approaches. Unlike the oscilloscope-based method, which continuously integrates the energy signal, the TOT method quantizes energy information into discrete clock cycles, leading to an inherent blurring effect.

For the CTR analysis, data within the FWTM value in each energy spectrum were utilized. To assess measurement error, the experiment was repeated three times, and the standard deviation of the FWHM results was calculated to determine both the average value and the associated error, as presented in figure 12(b). As mentioned in section 4.2, the time offset between the two timing channels was approximately 480 ps. For direct comparison, this offset was corrected by aligning the timing distributions to a common reference point at 0 ps.

The BGO TOF digitizer with NRBC achieved a CTR of 407 ± 8 ps FWHM, closely matching the 403 ± 14 ps FWHM obtained using the oscilloscope. In terms of FWTM values, the NRBC-based digitizer exhibited slightly better performance (1215 ± 78 ps) compared to the oscilloscope (1309 ± 31 ps). Meanwhile, the BC-based measurement resulted in a degraded CTR of 464 ± 26 ps FWHM and 1321 ± 86 ps FWTM. These results underscore the effectiveness of the NRBC and its DCL mechanism in preserving timing precision by mitigating early termination artifacts in TOT measurements.

Table 1 presents the resource utilization of the implemented BGO digitizer. The single-channel BGO digitizer, including the NRBC and DSM TDC, utilizes approximately 0.19% of the available LUTs and 0.06% of the available registers for the entire XC7VX485T-2FFG1761C device. This low FPGA resource usage facilitates seamless multi-channel scalability. When increasing the number of channels, the UART and main controller do not scale proportionally, allowing for the implementation of multiple channels with minimal overhead. Thus, the architecture is well-suited for large-scale PET detector systems. Furthermore, the power consumption of a dual-channel BGO digitizer system was measured at 0.219 W for dynamic power and 0.244 W for static power, including all peripheral components. This low-power consumption characteristic enables efficient system scalability, making it highly suitable for large-scale applications.

**Table 1.** Resource utilization of the single-channel BGO digitizer.

	Total resources	BGO digitizer (/channel)	UART TX & RX	Main controller
Slice LUTs	303 600	604	99	111
	(100%)	(0.19%)		

Slice Registers	607 200	342	61	195
	(100%)	(0.06%)		

### 4.5. Future work

The proposed FPGA-based BGO TOF digitizer demonstrates high precision in single-channel operation and is designed with scalability in mind, allowing seamless expansion to multi-channel TOF-PET detector systems. While this study establishes the fundamental feasibility of a single-channel module, its modular architecture facilitates straightforward integration into distributed processing frameworks.

Future work will focus on three key areas: improving energy resolution, enhancing timing performance, and optimizing scalability for multi-channel implementations. The current TOT-based energy measurement method, though efficient, introduces nonlinearity that limits energy resolution and obscures the Compton scattering region. A dual-threshold correction approach using two distinct *VT*_Energy_ values could mitigate this issue by improving alignment with oscilloscope-measured spectra (Grant and Levin 2014, Gaudin *et al* 2020). However, this approach requires additional comparators per channel, increasing system complexity. Future research will explore strategies to enhance energy resolution while maintaining resource efficiency.

Further improvements in timing measurement can be achieved through refined timewalk correction, such as dual-time-stamping with distinct *VT*_Timing_ thresholds to mitigate amplitude-related timing errors (Xie *et al* 2021). This is expected to enhance CTR performance, particularly under varying signal conditions. Additionally, further investigation is needed to better understand the observed trade-off between energy resolution and timing performance. Identifying the underlying mechanisms of this phenomenon will be crucial for optimizing both energy and timing resolution in future designs.

For multi-channel scalability, inter-channel delay correction will be critical to ensuring uniform timing across all channels. A calibration-based timing delay map can compensate for routing and component variations, maintaining synchronization in large-scale PET systems. Additionally, future efforts will focus on optimizing FPGA resource utilization and developing efficient communication protocols to support distributed signal processing in multi-channel architectures.

## 5. Conclusion

This study successfully developed and demonstrated the first FPGA-based TOF digitizer specifically designed for BGO-based TOF PET systems. This novel digitizer uniquely optimizes separate measurements of energy and timing data for both scintillation and Cerenkov photons generated in BGO, marking an important advancement in PET technology to accommodate the recently highlighted improvement in timing resolution achieved by detecting Cerenkov photons.

Key achievements include the implementation of a robust, NRBC for BGO energy signals and a resource-efficient DSM TDC, which achieved a superb average time bin resolution of approximately 6 ps. The digitizer exhibited effective TOT functionality for energy measurement and high accuracy and linearity in timing measurements. A direct comparison of the proposed BGO TOF digitizer with existing FPGA and non-FPGA solutions further highlights its practical advantages. Unlike oscilloscope-based digitizers, which offer high precision but are impractical for large-scale PET systems, our approach achieves comparable timing resolution while ensuring scalability and cost efficiency. Moreover, conventional FPGA-based designs typically rely on single-ended delay-line TDCs and standard binary counters, which introduce noise sensitivity and nonlinearity in TOT measurements. In contrast, DSM TDC mitigates timing errors due to PVT variations, while the NRBC ensures accurate energy measurement by filtering out premature terminations. These innovations collectively enhance the feasibility of BGO-based TOF PET by offering a resource-efficient, robust, and scalable digitization platform. Notably, when paired with 3 × 3 × 20 mm^3^ BGO crystals coupled to CHK-HD MT SiPMs, we achieved a CTR of 407 ps FWHM, demonstrating performance comparable to that measured using a high-performance oscilloscope.

## Data Availability

The data cannot be made publicly available upon publication because no suitable repository exists for hosting data in this field of study. The data that support the findings of this study are available upon reasonable request from the authors.
